# Comparison of conversion unicompartmental knee arthroplasties to primary and revision total knee arthroplasties: A gradient of complexity

**DOI:** 10.1016/j.jcot.2026.103430

**Published:** 2026-04-07

**Authors:** Johnathan R. Lex, Bahar Entezari, Sebastian Tomescu, Bheeshma Ravi

**Affiliations:** a Division of Orthopaedic Surgery, Department of Surgery, University of Toronto, Toronto, Canada; b Division of Orthopaedic Surgery, Sunnybrook Health Sciences Centre, Toronto, Canada; c ICES, Toronto, Canada; d Institute of Health Policy, Management & Evaluation, University of Toronto, Toronto, Canada

**Keywords:** Arthroplasty, Replacement, Knee, Reoperation, Prosthesis failure, Treatment outcome, Patient reported outcome measures, Matched-pair analysis

## Abstract

**Background:**

Unicompartmental knee arthroplasties (UKAs) are often revised to total knee arthroplasty (TKA), but concerns remain surrounding outcomes of UKA revision to TKA. The primary aim of this study was to compare revision rates of conversion UKA to TKA to matched cohorts of both primary and revision TKA procedures. Secondary aims were to compare implants characteristics, intraoperative, and functional outcomes.

**Methods:**

This was a single centre cohort study of all consecutive patients who underwent UKA conversion to TKA from 2012 to 2023. Patients undergoing conversion UKA with minimum two year follow-up were included and matched 1:1:1 to patients undergoing primary TKA and aseptic revision TKA, excluding periprosthetic fractures. Indications for surgery, surgical details, and postoperative outcomes, including revision rates, complications, and range of motion (ROM), were collected and compared between groups.

**Results:**

One hundred patients underwent conversion of a UKA to TKA that met inclusion criteria and were matched 1:1:1 to primary and revision TKAs (total n = 300). Post-matching, the mean age was 69.0 years and 75% were female. There was no statistically significant difference in revision rates between UKA to TKA (6.0%), primary TKA (1.0%) and revision TKA groups (14.0%), though reoperation rates were higher following UKA revision compared to primary TKA (p = 0.028). UKA conversion cases had longer operative times (103.0min vs. 72.7min, p < 0.001) and more frequent use of tibial augments (38% versus 0%, p < 0.001) and stems (56% versus 3%, p < 0.001) than primary TKA. UKA conversion demonstrated ROM similar to primary TKA (p = 1.000) but superior to revision TKA (p = 0.050).

**Conclusion:**

The findings of this study suggest conversion of UKA to TKA is more complex than primary TKA but less than revision TKA. Patients should be advised of higher reoperation rates compared to primary TKA, although similar functional outcomes. Surgeons should be prepared for increased implant complexity in these cases.

## 1 Introduction

An alternative to total knee arthroplasty (TKA), unicompartmental knee arthroplasty (UKA) is a surgical technique used for the treatment of end-stage osteoarthritis affecting only one compartment of the knee. Indications for UKA have changed considerably since its introduction, expanded to younger and overweight patients.^1^, ^2^, ^3^, ^4^, ^5^ This has led to a corresponding increase in the number of UKAs performed, up 590% between 2012 and 2022.^6^^,^^7^ Proponents for UKA have advocated for its use due to evidence suggesting lower rates of serious medical complications, mortality, a faster recovery, and similar to superior functional outcome.^8^, ^9^, ^10^, ^11^, ^12^ However, there continues to be concern regarding higher revision rates for UKA compared to TKA, with patients typically undergoing conversion of UKA to TKA if the former does not provide complete pain relief or if there is progression of arthritis in the other compartment(s).^13^^,^^14^

Conversion of a UKA to TKA poses difficulties, including addressing bone loss, soft-tissue balancing and maintaining the joint line.^15^^,^^16^ Such challenges may warrant conversion to a revision type of TKA, with use of stems, augments, thicker polyethylene, or constrained implants.^17^ Reported series of UKA to TKA conversions have reported a 30-40% rate of utilization of revision TKA components.^17^, ^18^, ^19^ Conversely, others have reported UKA to TKA conversions to not be technically difficult, endorsing outcomes comparable to primary TKA.^20^, ^21^, ^22^ There is very limited evidence directly comparing the outcomes of a conversion UKA-to-TKA to primary and revision TKA procedures.^23^

With increasing popularity of UKA, there is an anticipated rise in patients requiring conversion to TKA.^24^ Surgeons must thus be aware of the intraoperative challenges, implant requirements and postoperative outcomes to appropriately plan and counsel patients. It is known that revision TKAs have higher revision and complication rates than primary TKAs, however, the outcomes of TKA following conversion from UKA is still uncertain.^25^ Given the ongoing controversies surrounding the surgical complexity and outcomes of conversion UKA to TKA, our primary aim was to compare revision rates of these cases to matched cohorts of both primary TKA and revision TKA procedures. Secondary aims were to compare intraoperative outcomes, implants characteristics, and functional outcomes between these groups. The hypothesis of this study was that the added complexity of converting a UKA to a TKA would result in higher revision rates and worse outcomes compared to primary TKA but not revision TKA.

## 2 Methods

This was a consecutive single centre cohort study of all patients who underwent UKA conversion to TKA (referred to hereinafter as conversion UKA) from April 2012 to June 2023. Institutional research ethics board approval was granted prior to study commencement (Project ID: 6214). Patients were retrospectively identified from a prospectively maintained institutional arthroplasty database. All consecutive adult (>18 years) patients undergoing elective aseptic conversion of UKA to TKA were included. These conversion UKA patients were then matched 1:1:1 to patients undergoing primary TKA and first-time aseptic revision TKA with no prior UKA. Patients with revision TKA for infection or fracture were excluded as there is large heterogeneity in these diagnoses and procedures.^26^^,^^27^ A minimum two-year follow-up from the date of the index surgery was required, or to death.

Demographic and medical patient data at the time of index surgery including age, sex, body mass index (BMI), medical comorbidities, smoking history, American Society of Anesthesiologists’ (ASA) classification, and Charlson Comorbidity Index (CCI) classification. Indication for revision were recorded and grouped according to standardized definitions.^28^ Intraoperative data was collected including duration of surgery (defined as procedure start and finish times), and estimated blood loss. Details regarding the prosthesis implanted was also recorded including use of tibial and femoral stems, polyethylene thickness, patellar resurfacing, and implant level of constraint. Revisions were defined as any subsequent surgery with exchange of implant hardware, whereas reoperation was defined as a subsequent surgery that required no change of implants (excluding modular components). Other outcomes collected included preoperative and 1-year post-operative Western Ontario and McMaster Universities Osteoarthritis Index (WOMAC) scores, range of motion (ROM), fixed flexion contracture, and length of stay. WOMAC scores evaluate pain, physical function and stiffness in patients with hip and knee arthritis and were collected at each timepoint. ROM was measured using goniometers in clinic by staff surgeons or advanced practice physiotherapists. Duration of follow-up from index surgery was documented for all patients.

### 2.1 Surgical technique

Conversion UKA procedures were performed by 11 fellowship trained arthroplasty surgeons. Existing midline, paramedian, or curved medial scars were used for skin incisions wherever possible, followed by a standard medial parapatellar arthrotomy. Hardware from prior UKA or TKA was removed using osteotomes and thin reciprocating saw blades. Upon visualization of the patellar articulating surface, patellar resurfacing was performed if significant patellar wear was noted. Otherwise, patelloplasty and lateral facetectomy was undertaken to ensure a smooth articulating surface. Manual instrumentation was used to guide bone resection and implant positioning for all cases. Metal tibial and femoral augments were used if significant bone loss (>2-3 mm) was noted during component trialing, otherwise small contained and uncontained defects were filled with cement augments. Likewise, tibial and femoral stems were used if poor metaphyseal bone stock was noted at time of surgery necessitating additional implant stability from diaphyseal fixation. Choice of cemented or uncemented stems was determined by bone quality, existing hardware and surgeon preference. Trial implants were used to assess joint stability and ROM, and to determine the need for any additional releases. Definitive implants were all implanted using antibiotic-impregnated cement.

### 2.2 Statistical analysis

Propensity score matching was performed using nearest-neighbour matching without replacement to match patients from the conversion UKA group to primary and revision TKA patients (in a ratio of 1:1:1) based on age, sex, and BMI. A caliper width of 0.2 standard deviations of the propensity score was utilized. There were no differences based on standardized mean differences (values < 0.1) following matching. Differences in continuous data were assessed using unpaired Students t-test and categorical data using Pearson's Chi-squared test or Fisher's exact test where applicable. When comparing differences in continuous outcomes between the three procedure groups, one-way analysis of variance (ANOVA) with post hoc Tukey HSD test was utilized. Hazard ratios (HR) were calculated using cox proportional hazards model to compare implant survival and reoperation between groups. Kaplan-Meier survival analysis with log-rank test was performed for evaluating differences in implant revision and reoperation rates. Subgroup analysis by implant type within the conversion UKA group was also performed to determine the association between certain implant characteristics (use of augments, stems and constraint) and outcome. Statistical significance for all analyses was set at α = 0.05. Analysis was performed using Stata software (Version 16.1, StataCorp, USA).

## 3 Results

A total of exactly 100 patients underwent a conversion UKA during the study period. These were all successfully matched to a cohort of primary TKA and revision TKA procedures, totalling 300 patients. Following matching there was no difference in age, sex, BMI, diabetes or smoking status, ASA classification or Charlson comorbidity index (Table 1). Mean follow-up following revision UKA-to-TKA, primary and revision TKA was 6.8 years (SD: 3.2, range: 1.7 to 12.2 years), 6.1 years (SD: 3.1, range: 2.2 to 12.0 years) and 6.5 years (SD: 3.0, range: 2.3 to 12.2 years), respectively (p > 0.05).

**Table 1:** Baseline demographic features and medical comorbidity indices of all three groups.

	Revision UKA (N = 100)	Primary TKA (N = 100)	Revision TKA (N = 100)	p-value^a^
**Age, mean (SD)**	69.0 (10.2)	69.3 (10.5)	69.2 (10.4)	0.972
**Females, N (%)**	75 (75.0)	75 (75.0)	75 (75.0)	1.000
**BMI, mean (SD)**	30.8 (6.5)	30.7 (5.7)	31.2 (6.8)	0.864
**BMI ≥ 35, N (%)**	23 (23.0)	23 (23.0)	23 (23.0)	1.000
**Diabetes, N (%)**	15 (15.0)	17 (17.0)	18 (18.0)	0.845
**Smoker, N (%)**	13 (13.0)	7 (7.0)	5 (5.0)	0.311
**ASA Classification, N (%)**				
**I**	0 (0.0)	1 (1.0)	0 (0.0)	0.655
**II**	30 (30.0)	34 (34.0)	30 (30.0)	
**III**	67 (67.0)	64 (64.0)	66 (66.0)	
**IV**	3 (3.0)	1 (1.0)	4 (4.0)	
**Charlson Comorbidity Index, N (%)**				
**0**–**1**	16 (16.0)	16 (16.0)	12 (12.0)	0.916
**2**–**3**	56 (56.0)	54 (54.0)	58 (58.0)	
**>3**	28 (28.0)	30 (30.0)	30 (30.0)	

a Analysis of variance test. ASA = American Society of Anaesthesiologists; BMI = Body Mass Index.

In the conversion UKA cohort, 97.0% were of the medial compartment, 73.0% mobile bearing, and 82.0% cemented. The indications for conversion UKA to TKA and the revision TKA cohort are listed in Table 2. All primary TKA cases were performed for degenerative (n = 97, 97.0%) or inflammatory arthritis (n = 3, 3.0%). Mean time between the primary UKA and conversion to TKA was 8.8 years (SD: 5.5 years) and was 6.8 years (SD: 6.2 years) for the revision TKA group.

**Table 2:** Reason for revision for revision UKA to TKA group and revision TKA group.

Reason for revision, N (%)	Revision UKA	Revision TKA
New Arthritis in Other Compartment	37 (37.0)	-
Aseptic Loosening	18 (18.0)	24 (24.0)
Instability	14 (14.0)	30 (30.0)
Malpositioning	12 (12.0)	28 (28.0)
Bearing Wear/Osteolysis	8 (8.0)	5 (5.0)
Pain of Unknown Origin	6 (6.0)	4 (4.0)
Stiffness	3 (3.0)	9 (9.0)
Implant Dissociation	2 (2.0)	-

### 3.1 Revision and reoperation

**Fig. 1:**
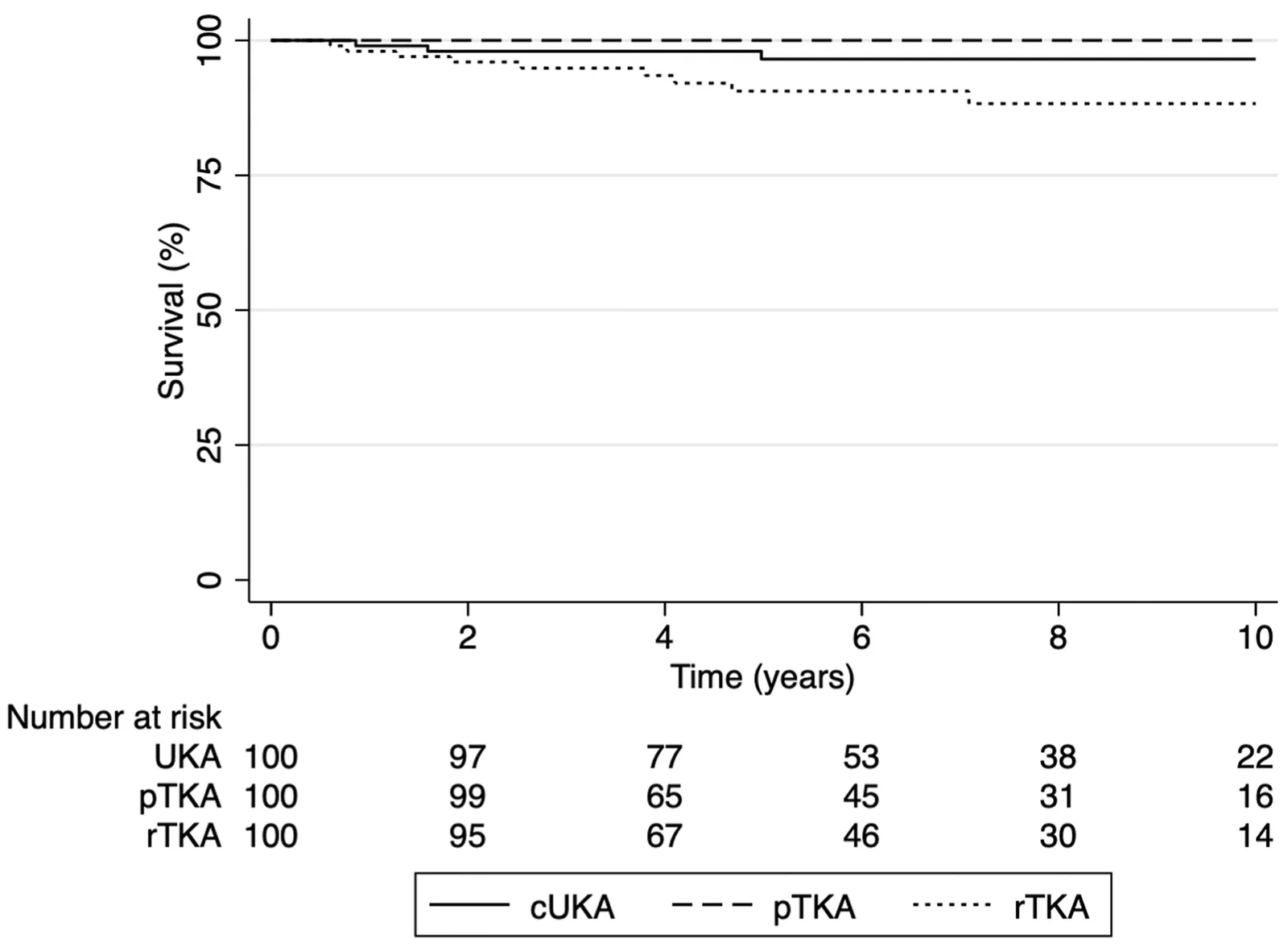
10-year Kaplan-Meier curve for all-cause revision-free survival (log-rank p > 0.05). cUKA = Conversion unicompartmental knee arthroplasty to total knee arthroplasty; pTKA = Primary total knee arthroplasty; rTKA = Revision total knee arthroplasty.

**Fig. 2:**
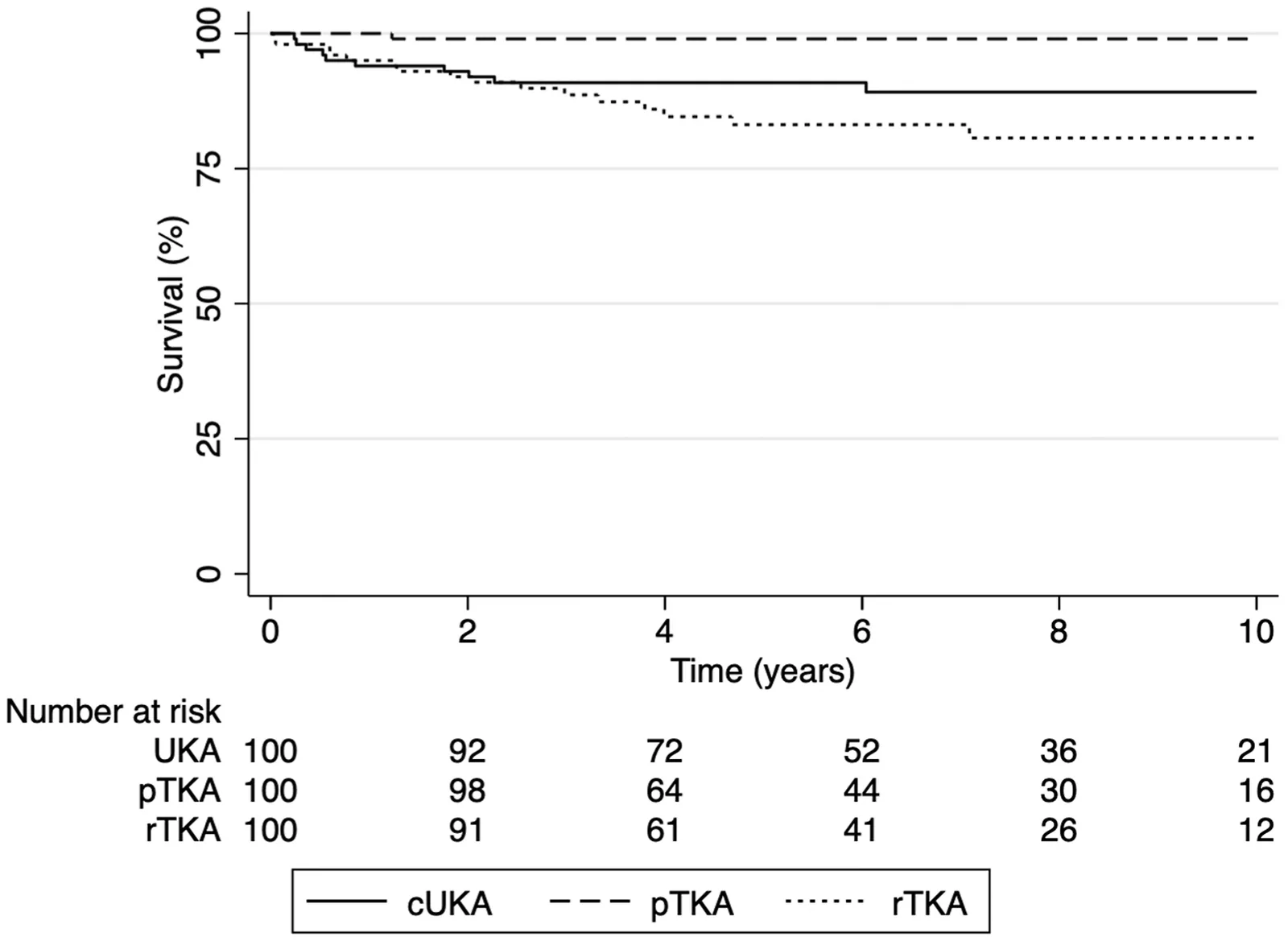
10-year Kaplan-Meier survival curve for all-cause reoperation. (log-rank, p = 0.028 between pTKA and conversion UKA; log-rank, p = 0.213 between conversion UKA and rTKA). cUKA = Conversion unicompartmental knee arthroplasty to total knee arthroplasty; pTKA = Primary total kneearthroplasty; rTKA = Revision total knee arthroplasty.

The revision rate of the conversion UKA group was 6.0%, and 1.0% and 14.0% in the primary and revision TKA, respectively (Table 3). There was no statistically significant difference in revision rates between the conversion UKA group and the primary or revision TKA group (p > 0.05, HR not calculatable due to low revision rates) (Fig. 1). There was a higher risk of reoperation in the conversion UKA group compared to the primary TKA group (HR = 10.08, 95% CI: 1.29-78.78, p = 0.028). There was also no significant difference in reoperation rates between the conversion UKA and the revision TKA groups (HR = 0.61, 95% CI: 0.27-1.33, p = 0.213) (Fig. 2). Reasons for revision in the UKA to TKA group included stiffness (n = 2, 2.0%), instability (n = 1, 1.0%), PJI (n = 1, 1.0%), and patellofemoral joint resurfacing (n = 1, 1.0%). There was only one revision in the primary TKA group, which was for patellar instability. Re-revision in the revision TKA group was for instability (n = 6, 6.0%), aseptic loosening (n = 2, 2.0%), pain from prominent components (n = 2, 2.0%), PJI (n = 1, 1.0%), patellofemoral joint resurfacing (n = 1, 1.0%), stiffness (n = 1, 1.0%), and patellar instability (n = 1, 1.0%).

**Table 3:** Postoperative revision and functional outcomes for each group.

	Revision UKA (N = 100)	Primary TKA (N = 100)	p-value^a^	Revision TKA (N = 100)	p-value^b^
Revision, N (%)	6 (6.0)	1 (1.0)	0.054	14 (14.0)	0.059
Reoperation, N (%)	10 (10.0)	1 (1.0)	0.005	15 (15.0)	0.285
Length of Stay (days), N (SD)	2.9 (1.3)	2.7 (2.2)	0.743	3.2 (1.9)	0.502
Range of Motion >90^o^, N (%)	98 (98.0)	98 (98.0)	1.000	91 (91.0)	0.050
Flexion Contracture, N (%)					
None	83 (83.0)	71 (71.0)	0.130	73 (73.0)	0.137
0^o^ to 15^o^	16 (16.0)	27 (27.0)		21 (21.0)	
>15^o^	1 (1.0)	2 (2.0)		5 (5.0)	
1-year WOMAC Score, mean (SD)^a^	23.7 (18.2)	17.3 (14.8)	0.169	28.3 (19.1)	0.410
Change in WOMAC Score, mean (SD)^b^	22.0 (16.5)	33.9 (20.5)	0.008	19.3 (20.2)	0.781

aTukey HSD between Revision UKA group and primary TKA group.

bTukey HSD between Revision UKA group and revision TKA group.

a Patients: UKA n = 45, primary TKA = 53, revision TKA = 45.

b Patients: UKA n = 44, primary TKA = 53, revision TKA = 44 WOMAC = Western Ontario and McMaster Universities Osteoarthritis Index.

There was no difference in revision rates in the UKA to TKA group with the use of tibial stems, femoral stems, tibial augments or femoral augments. There was also no difference in revision rates between cemented or uncemented UKAs or whether it was a revised medial or lateral compartment UKA. However, the interpretation of these analyses are limited by the low number of failures.

### 3.2 Intraoperative outcomes & implants

There was a significant difference in duration of surgery between all three groups, with the primary TKA having the shortest duration of surgery, followed by the conversion UKA group, and finally the revision TKA group (p < 0.001) (Table 4).

**Table 4:** Intraoperative outcomes and knee components implanted for each group.

	Revision UKA (N = 100)	Primary TKA (N = 100)	p-value^a^	Revision TKA (N = 100)	p-value^b^
Duration of Surgery (min), mean (SD)	103.0 (30.4)	72.7 (17.7)	<0.001	134.4 (47.5)	<0.001
Estimated Blood Loss (ml), mean (SD)	110.4 (134.3)	64.0 (92.7)	0.134	182 (246)	0.009
Femoral Stem, N (%)	7 (7.0)	1 (1.0)	0.030	73 (73.0)	<0.001
Tibial Stem, N (%)	56 (56.0)	3 (3.0)	<0.001	90 (90.0)	<0.001
Femoral Augment, N (%)	3 (3.0)	0 (0.0)	0.081	57 (57.0)	<0.001
Tibial Augment, N (%)	38 (38.0)	0 (0.0)	<0.001	29 (29.0)	0.178
Patellar Resurfacing, N (%)	0 (0.0)	1 (1.0)	0.316	6 (6.0)	0.013
Polyethylene Thickness (mm), mean (SD)	12.4 (2.3)	11.1 (1.7)	<0.001	14.0 (3.0)	<0.001
Knee Constraint, N (%)					
Cruciate-Retaining (CR)	6 (6.0)	5 (5.0)	0.554	0 (0.0)	<0.001
Posterior Stabilized (PS)	88 (88.0)	92 (92.0)		51 (51.0)	
Constrained Condylar Knee (CCK)	6 (6.0)	3 (3.0)		49 (49.0)	

a Tukey HSD between Revision UKA group and primary TKA group.

b Tukey HSD between Revision UKA group and revision TKA group.

There was a significant difference between all three groups in requirement for femoral and tibial stems, with primary TKA requiring them the least frequently and revision TKA the most. This was similar for femoral augments. However, tibial augments were more commonly required in the conversion UKA to TKA group than in the revision TKA group (Table 4). There was no difference in level of polyethylene bearing constraint between primary and conversion UKA cases, however, there was a significant difference between conversion UKA and revision TKA cases, with revision TKA cases more commonly requiring a varus-valgus constrained level of constraint (Table 4).

### 3.3 Functional outcomes

There was no difference in 1-year postoperative WOMAC scores between conversion UKA and the other two groups, however, the primary TKA group had a greater mean improvement in WOMAC scores than the revision UKA group from pre-operative to 1-year postoperative (p = 0.008). At 1-year, the mean conversion UKA extension was 1.3° (SD: 3.2), and 119.5° (SD: 10.9) of flexion. Compared to revision TKA, the conversion UKA group had higher rates of patients achieving an arc of knee ROM of greater than 90° (p = 0.050). There was no difference in average patient length of stay (Table 3).

## 4 Discussion

This study sought to compare the outcomes of converting a UKA to a TKA to primary and revision TKAs. The present study was one of the largest matched comparisons evaluating conversion UKA-to-TKA alongside both primary and revision TKA cohorts, allowing direct assessment of surgical complexity and outcomes. The most important finding of this study was that the complexity of conversion UKA to TKA as assessed by revision rates, functional outcomes, complexity of implants, and intraoperative difficulty, is distinct, and lies between primary and revision TKA. Conversion UKAs can expect higher reoperation rates compared to primary TKAs. The differences identified, while not statistically significant, are clinically meaningful, likely reflective of the study sample size and low outcome event rate. These are important considerations when discussing the options of either UKA or TKA with patients. Clinicians and researchers should focus on optimal patient selection for UKA to minimize required conversions to TKA.

With regards to the primary outcome of this study, there was no statistically significant difference in revision between conversion UKA to TKA and either of the other groups. Though the primary TKA group had the lowest revision rate, 1%, compared to 6% and 14% in the conversion UKA and revision TKA groups, respectively. The reoperation rate for the conversion UKA group was significantly greater than in the primary TKA cohort. Overall, this raises concern that the survivorship of TKAs that have been converted from a UKA is not as reliable as a primary TKA in a native knee. On the other hand, conversion UKAs performed favourably compared to revision TKA which may be an important consideration when deciding between primary UKA and TKA. This is particularly true for younger patients that are more likely to require an aseptic revision UKA or TKA in their lifetime.^29^ This is an important clinical finding to discuss when pre-operatively counselling patients on the risks and outcomes of both UKA and conversion UKA procedures.

Reasons for re-revision between groups were relatively similar, including for PJI, however, the revision TKA group was more likely to be re-revised for instability. This is likely secondary to the larger degree of soft-tissue dissection required for removal of TKA components compared to UKA components and lower use of hinge-level constraint. Contrary to previous studies, no TKAs in the conversion UKA group were revised for aseptic loosening.^30^ This may be explained by the relatively high utilization rate of tibial stems and augments in this cohort.^17^^,^^30^

The mean operative time was greater in the converted UKA group compared to the primary TKA group but shorter than the revision TKA group. Similarly, there was greater blood loss in the revision TKA group than the revision UKA group. While there was no statistically significant difference between the primary and revision UKA group, there was an observed trend between these groups (64.0 ml vs. 110.4 ml, respectively). These findings are similar to previous reports comparing revision UKAs to primary and revision TKA.^23^^,^^30^ There is evidence that longer operative times and blood loss are associated with perceived intraoperative difficulty and higher complications rates.^31^, ^32^, ^33^ Therefore, when performing conversion UKA to TKA cases, surgeons should schedule additional time compared to primary TKA cases and be prepared for more intraoperative difficulty.

Greater use of both femoral and tibial augments and stems likely contributed to higher operative times in the conversion TKA group than the primary TKA group. Conversely, compared to revision TKA, there was a lower utilization of femoral and tibial stems and femoral augments. Interestingly, there was no difference in utilization of tibial augments between conversion UKA to TKA and revision TKA cases as roughly 40% of conversion UKA cases required tibial augments, similar or greater than previously published series.^23^ This finding highlights the prevalence of tibial bone defects and strategies to acquire adequate fixation. Care must be taken to recreate a stable tibial platform (either with augments or bone graft) and/or metaphyseal or diaphyseal fixation with the use of cones or stems, respectively, with the goal of achieving adequate fixation in 2 of 3 tibial zones during conversion UKA to TKA cases. Femoral augments and stems were used in just 3% and 7% of cases, respectively, whereas tibial augments and stems were used in 38% and 56%, respectively. The incorporation of a stem or augment aimed to provide structural stability and safeguard the reconstruction of bony defects by improving strain distribution within the proximal tibia. As there were no early to mid-term revisions for aseptic loosening in this series, we believe this to be an effective strategy, acknowledging that the specific category of bone loss was not recorded. Surgeons should ensure they have the appropriate implants available, greater than the requirements for a primary TKA, and appropriately allocated operative time, when performing these cases. Given the technical complexity, increased operative time, and need for advanced reconstruction techniques, greater resource utilization and support is required to complete these cases than for primary TKAs.

In this present study, 1-year postoperative WOMAC scores were lowest among patients undergoing primary TKA (lower score representing better function) and highest among patients undergoing revision TKA, although there were no statistically significant differences in 1-year postoperative WOMAC scores between conversion UKA patients and primary TKA patients or revision TKA patients. These findings suggest that patients undergoing conversion UKA, primary TKA, and revision TKA have comparable pain, stiffness, and functional outcomes. In contrast to our study, in their 2015 investigation of 48 conversion UKAs matched 1:1:1 to primary TKAs and revision TKAs, Lunebourg et al. found postoperative Knee Society knee scores and Knee Society function scores of patients undergoing conversion UKA to be worse than those of patients undergoing primary TKA and similar to those of patients undergoing revision TKA.^23^ Additionally, Lunebourg et al. found ROM in UKA to TKA patients to be similar to revision TKA patients, while here we demonstrate ROM among UKA to TKA patients is superior to revision TKA patients. Järvenpää et al. demonstrated that conversion UKA patients had poorer WOMAC scores compared to primary TKA patients; comparison to revision TKA patients was not made.^34^ Conversely, other studies have demonstrated that functional outcomes following conversion UKA to TKA are comparable to those following primary TKA.^35^ In their 2018 study, Lombardi et al. found Knee Society clinical scores after conversion UKA to TKA to be inferior compared to primary TKA, but functional scores to be equivalent.^11^ Heterogeneity in the functional outcomes reported in the literature may in part be attributed to reason for conversion of UKA to TKA. In a 2013 study comparing patients undergoing UKA to TKA for unexplained pain versus for defined cause for pain, patients with unexplained pain had poorer outcomes on the Oxford knee score and visual analogue scale.^36^ Additional factors which may contribute to the heterogeneity in the literature include differences in surgical techniques, patient selection criteria, and postoperative rehabilitation protocols across studies.

While this study is a relatively large, consecutive series comparing three matched groups of knee replacement, it has various limitations. One limitation is that the surgeries in this cohort were performed at one centre by 11 different experienced arthroplasty surgeons. While the number of surgeons adds to the generalizability of the study, it blurs the indications for certain implant choices, such as the levels of polyethylene constraint, use of augments and stems. Despite this, no difference in revision was found by use of augments or stems. Another main limitation to this study is the retrospective nature of the study. This may add the risk of selection bias, particularly to the primary and revision TKA subgroups, however, this risk was mitigated by utilizing propensity score matching. We also aimed to mitigate this risk by matching by the conversion UKA and revision TKA groups by aseptic failures and excluding periprosthetic fractures. It should be noted that preoperative and 1-year WOMAC scores were only available for approximately 50% of study participants. Sensitivity analyses were performed to evaluate for any potential bias between patients with and without completed 1-year WOMAC scores and were not found, nevertheless these results should still be interpreted with caution due to residual confounding. Finally, these results represent follow-up at the mid-term range and longer follow-up information is required, particularly to fully elucidate the impact of augments and stems on aseptic loosening.

Future research should focus on multicentre registry-based analyses with larger event numbers to better evaluate survivorship differences between procedures. Prospective collection of patient-reported outcome measures is required to reduce missing data bias. Longer-term follow-up will also be important to determine whether increased use of stems and augments influence the rates of late aseptic loosening. Additionally, in recent years, the evaluation of robotic-assisted conversions of UKA to TKA are more performed and the impact of these technologies on patient outcomes should be carefully evaluated.

## 5 Conclusions

The conversion of a UKA to a TKA is more complex than performing a primary TKA but less so than revision TKA. Patients should be warned that these cases have higher revision and reoperation rates than primary TKAs, however, overall functional outcomes appear similar to primary TKA. Typically, a standard posterior-stabilized implant can be utilized for this conversion, however, surgeons should be prepared that these cases may require stems and augments on the tibial side.

## Guardian/patient consent

Not applicable.

## Credit statement

JRL: Conceptualization, methodology, visualization, writing – original draft, BE: Validation, investigation, formal analysis, writing – review & editing, ST: Resources, data curation, writing – review & editing BR: data curation, writing – review & editing, supervision.

## Ethical approval

Institutional research ethics board approval was granted prior to study commencement (Project ID: 6214).

## Human ethics

Ethics approval was received following local institutional review board review (Project ID: 6214).

## Funding

No funding was used to conduct this study.

## Declaration of competing interest

Each author certifies that he or she has no commercial associations (e.g. Consultancies, stock ownership, equity interest, patent/licensing arrangements, etc.) that might pose a conflict of interest in connection with the submitted article.

## Abbreviations:

TKA: Total knee arthroplasty
UKA: Unicompartmental knee arthroplasty
ROM: Range of motion
WOMAC: Western Ontario and McMaster Universities Osteoarthritis Index
ASA: American Society of Anesthesiologists
BMI: Body mass index
ANOVA: Analysis of variance
